# Diabetes mellitus, malnutrition, and sarcopenia: The bond is not explained by bioelectrical impedance analysis in older adults

**DOI:** 10.25122/jml-2023-0173

**Published:** 2023-08

**Authors:** Francesco Salis, Francesca Zanda, Federica Cherchi, Benedetta Puxeddu, Luisa Sanna, Chiara Scudu, Silvia Serreli, Lorenzo Stanisci, Efisio Cossu, Antonella Mandas

**Affiliations:** 1 Department of Medical Sciences and Public Health, University of Cagliari, Cagliari, Italy; 2 Department of Medicine, Surgery, and Pharmacy, University of Sassari, Sassari, Italy; 3 University Hospital Azienda Ospedaliero-Universitaria of Cagliari, Cagliari, Italy

**Keywords:** Bioelectrical Impedance Analysis (BIA), Diabetes Mellitus (DM), elderly, malnutrition, sarcopenia, AC: Arm Circumference, ASM: Appendicular Skeletal Muscle Mass, BIA: Bioelectrical Impedance Analysis, BMI: Body Mass Index, C.I.: Confidence Interval, CC: Calf Circumference, DM: Diabetes Mellitus, ECW: Extra-Cellular Water, HbA1c: glycated hemoglobin, HDL: High-Density Lipoproteins, LDL: Low-Density Lipoproteins, MM: Total Muscle Mass, MNA: Mini Nutritional Assessment, SD: Standard Deviation, TBW: Total Body Water, WC: Waist Circumference

## Abstract

As people age, their risk of diabetes mellitus (DM) and sarcopenia increases due to the decline in muscle mass and strength. Bioelectrical impedance analysis (BIA) is a method used to detect changes in body composition. The primary aim of the study was to determine the distribution of BIA variables among a group of non-DM people and two groups of patients with controlled and uncontrolled DM. The secondary aim was to establish the independent association between BIA-derived data, lipidic assets, and the prevalence of metabolic syndromes with DM. This study included a total of 235 participants who were categorized into three groups based on the presence of diabetes mellitus (DM) and their glycated hemoglobin (HbA1c) levels: non-DM, controlled DM (HbA1c≤7.0%), and uncontrolled DM (HbA1c>7.0%). Waist circumference (p=0.005), bone (p<0.001), muscular (p<0.001), and appendicular skeletal mass (p<0.001) were lower in the non-DM group, while sarcopenic risk (p<0.001), total cholesterol (p<0.001), and LDL (p<0.001), were higher. Grip strength (p<0.001), visceral fat (p=0.01), and phase angle (p=0.04) were significantly lower in non-DM than uncontrolled DM patients, as well as the number of drugs taken (p=0.014). A multivariate analysis highlighted that LDL (coefficient -0.006, p=0.01) was negatively associated, while bone mass (coefficient 0.498, p=0.0042) was positively associated with DM uncontrol. Our study shows that BIA may not be the ideal tool for distinguishing between elderly individuals with and without DM, as it can be affected by numerous covariates, including potential differences in glucometabolic and cardiovascular control.

## INTRODUCTION

The aging process affects not only the physical body but also the social, psychological, and economic dimensions [1, 2]. As current trends indicate a continuous exponential growth in the elderly, the global population will be more likely to suffer from comorbidities, such as neurocognitive disorders [3], cardiorespiratory syndromes [4-6], psychiatric disorders [7], falls and fractures [8-10], which in turn can lead to an increased risk of frailty and mortality [11-14]. A multidimensional evaluation [15, 16] is an essential tool to assess this particular population, which is dealing with various multisystemic comorbidities [17-20] and the potential risks associated with polypharmacy [21, 22]. An early evaluation of cognitive-affective status [23-26], functional abilities [27-29], and nutritional and metabolic status [30-32] can help the patient have better long-term outcomes [33-35]. Among the mentioned multimorbidity, metabolic pathologies are of particular interest. Diabetes mellitus (DM) has been extensively studied in the literature and is associated with frailty [5], sarcopenia [36], and increased mortality [37]. Sarcopenia is characterized by reduced muscle mass and reduced strength (with or without decreased physical performances) and is associated with low quality of life and other chronic conditions [36, 38, 39]. Many studies focus on bioelectrical impedance analysis (BIA) [36, 40, 41], a useful tool to show the aging process in its multidimensional context and its importance in understanding the implications of sarcopenia and metabolic issues on human composition. However, even if its clinical and scientific role is clearly established, it is equally clear that it presents some inaccuracies, depending on specific parameters assessed [42], mathematical models [43], or, to the best of our knowledge, the absence of BIA validation in DM sarcopenic population [36]. Moreover, scientific literature usually focuses on younger and less comorbid populations than real-world ones [44].

### Aims and objectives

The primary aim of the study was to determine the different distribution of bioelectrical impedance variables among a group of individuals without DM and two groups of individuals with controlled and uncontrolled DM. The secondary aim of the study was to establish the independent association between BIA-derived data, lipidic assets, and the prevalence of metabolic syndromes in individuals with DM.

## MATERIAL AND METHODS

### Design of the study

This observational cross-sectional study included subjects consecutively evaluated at the Geriatric Outpatient Service of the University Hospital of Monserrato, Cagliari, Italy, between February and October 2021.

### Sample size

Considering a confidence level of 95%, a confidence interval of 5%, a standard deviation (SD) of 0.5, a Z-score (z) of 1.96, and an error margin (e)of 7%, the final sample (N) was calculated to be at least 196 subjects, according to the formula:


$$N=\frac{z^{2}*SD\left(1-SD\right)}{e^{2}}$$

### Inclusion and exclusion criteria

The inclusion criteria for this study encompassed individuals aged 65 years or older who had undergone anthropometric assessment, nutritional evaluation, sarcopenic screening, and bioelectrical impedance analysis (BIA). In contrast, exclusion criteria included individuals younger than 65, those with pacemakers or other implanted devices, individuals with static-dynamic instability, and those who did not provide informed consent. A total of 235 subjects met the specified inclusion criteria.

### Assessment

The enrolled subjects were evaluated with the following:

- Mini Nutritional Assessment (MNA) for nutritional assessment [45, 46]
- Strength, Assistance in walking, Rise from a chair, Climb stairs, and Falls (SARC-F) for the assessment of sarcopenic risk [47]
- Anthropometric measures [Body Mass Index (BMI), Waist Circumference (WC), Calf Circumference (CC), Arm Circumference (AC)]
- Muscle strength evaluation was conducted using a dynamometer
- BIA [40], which included the assessment of subcutaneous and visceral fat, bone mass, total (MM) and appendicular skeletal muscle mass (ASM), total (TBW) and extra-cellular water (ECW), phase angle, and metabolic age
- Measurement of blood lipid levels, including total cholesterol, triglycerides, high-density lipoproteins (HDL), and low-density lipoproteins (LDL).
- Determination of glycated hemoglobin (HbA1c) levels in patients with diabetes.

These tests and assessments were carried out by trained geriatricians in an outpatient setting. The diagnosis of sarcopenia was based on quantitative measurements of muscle mass, strength, and functional aspects.

### Statistical analysis

Variables were expressed as means and standard deviations (SDs) or percentages (%), where appropriate. Analysis of variance (ANOVA) was used to study the variance of the variables among the groups. Scheffé test was used for post-hoc analysis. Multivariate analysis was performed with a multiple regression – stepwise (p-values>0.1 were excluded by the model). Its results were expressed as coefficients and standard errors. The results are reported indicating p-values in reference to 95% C.I. MedCalc software (Version 20.218, Ostend, Belgium) was used for statistical analysis.

## RESULTS

**Figure 1:**
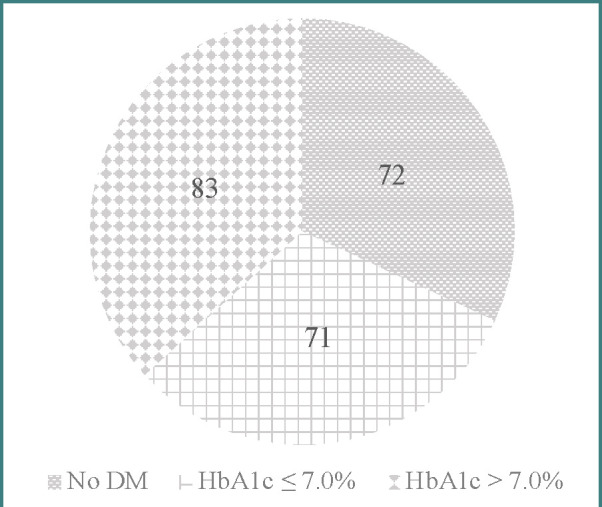
Groups classification

The study included 235 community-dwelling individuals aged 65 years or more. The characteristics of the sample are summarized in Table 1. Among these participants, 163 subjects (69.4%) had diabetes mellitus (DM group), with 3 having type-1 DM. The other 72 subjects, of whom 51 (70.8%) were women, made up the non-DM group. Nine patients with DM were excluded from further analysis due to their missing HbA1c values. The final DM group consisted of 154 subjects, of whom 78 (50.6%) were women. The DM group was further divided according to HbA1c levels in controlled DM (HbA1c≤7.0%, 83 subjects) and uncontrolled DM (HbA1c>7.0%, 71 subjects) (Figure 1).

**Table 1:** Sample characteristics

Variable	MIN	MAX	Mean	SD
Age (years)	65	93	76.7	6.9
BMI (kg/m^2^)	16.6	51.9	27.9	5.6
WC (cm)	64	136	96.7	13.1
CC (cm)	18	45.4	34.3	3.7
AC (cm)	18	42	28.6	3.9
Grip Strength (kg)	2	53	23.5	10.3
MNA	13.5	30	24.6	3
SARC-F	0	9	2.4	2.6
**BIA**				
Subcutaneous Fat (kg)	2.4	62.5	22.1	9.7
Visceral Fat (kg)	3	27	10.3	4.2
Bone Mass (kg)	1.4	3.6	2.4	0.4
MM (kg)	16.8	70	43.9	8.6
ASM (kg)	11.3	32.9	18.6	3.9
TBW (%)	31.6	68.7	48.8	6.5
ECW (%)	14.4	54	46.6	4.1
Phase Angle (degrees)	2.6	6.8	4.6	0.7
Metabolic Age (years)	48	90	68.8	10.5
**Blood Lipids**				
Total Cholesterol (mg/dl)	79	323	176.9	38.8
HDL (mg/dl)	20	102	56.3	13.6
Triglycerides (mg/dl)	38	278	99.3	44.8
LDL (mg/dl)	33.2	214.2	100.3	33.7
**Comorbidities**	%			
Hypertension	76.9			
Dyslipidemia	79.6			
Metabolic Syndrome	68.1			
Sarcopenia	13.2			

SD, Standard Deviation; BIA, Bioelectrical Impedance Analysis; BMI, Body Mass Index; WC, Waist Circumference; CC, Calf Circumference; AC, Arm Circumference; MNA, Mini Nutritional Assessment; MM, total Muscle Mass; ASM, Appendicular Skeletal Muscle Mass; TBW, Total Body Water; ECW, Extra-Cellular Water; HDL, High-Density Lipoproteins; LDL, Low-Density Lipoproteins

The analysis of variance showed that BMI (p=0.298), CC (p=0.073), AC (p=0.081), subcutaneous fat (p=0.253), TBW (p=0.932), ECW (p=0.695), metabolic age (p=0.378) presented nonsignificant differences among the groups. Post-hoc analysis demonstrated that phase angle and HDL, although having significant p-values (0.040 and 0.033, respectively), did not show differences among the three groups (Table 2).

**Table 2:** ANOVA and post-hoc analysis

Variable	Non-DM (n. 72)		Controlled DM (n. 83)		Uncontrolled DM (n. 71)		ANOVA	Scheffé
	Mean	SD	Mean	SD	Mean	SD	p-value	different from
**Age (years)**	78.9	6.1	75.1	6.5	76.4	7.5	0.002	1 *vs* 2
								2 *vs* 1
								-
**BMI (kg/m^2^)**	27.1	6.4	27.9	4.7	28.5	5.2	0.298	-
								-
								-
**WC (cm)**	92.6	13.4	97.6	12.0	99.4	12.9	0.005	1 *vs* 3
								-
								3 *vs* 1
**CC (cm)**	33.5	4.4	34.6	3.1	34.8	3.3	0.073	-
								-
								-
**AC (cm)**	27.7	4.5	29.0	3.6	28.9	3.4	0.081	-
								-
								-
**Grip strength (kg)**	19.1	8.2	26.1	9.9	25.6	11.2	<0.001	1 *vs* 2 and 3
								2 *vs* 1
								3 *vs* 1
**MNA**	23.5	3.1	25.5	2.7	25.1	2.9	<0.001	1 *vs* 2 and 3
								2 *vs* 1
								3 *vs* 1
**SARC-F**								1 *vs* 2 and 3
	3.7	2.6	1.5	2.1	1.9	2.4	<0.001	2 *vs* 1
								3 *vs* 1
**HbA1c (%)**								-
	-	-	6.3	0.5	8.2	1.3	<0.001	2 *vs* 3
								3 *vs* 2
**Subcutaneous fat (kg)**								-
	20.4	10.8	22.4	8.9	22.9	9.1	0.253	-
								-
**Visceral fat (kg)**								1 *vs* 3
	9.2	4.3	10.3	3.6	11.2	4.1	0.010	-
								3 *vs* 1
**Bone mass (kg)**								1 *vs* 2 and 3
	2.2	0.4	2.4	0.4	2.5	0.4	<0.001	2 *vs* 1
								3 *vs* 1
**MM (kg)**								1 *vs* 2 and 3
	40.6	8.2	44.8	8.4	46.7	8.6	<0.001	2 *vs* 1
								3 *vs* 1
**ASM (kg)**								1 *vs* 2 and 3
	17.1	3.5	19.1	3.6	19.7	4.2	<0.001	2 *vs* 1
								3 *vs* 1
**TBW (%)**								-
	49.1	6.4	48.7	6.6	48.8	6.3	0.932	-
								-
**ECW (%)**								-
	46.5	4.8	46.9	3.0	46.6	2.8	0.695	-
								-
**Phase angle (degrees)**								-
	4.4	0.7	4.7	0.8	4.7	0.7	0.040	-
								-
**Metabolic age (years)**								-
	68.6	9.7	67.4	10.4	69.7	11.1	0.378	-
								-
**Total cholesterol (mg/dl)**								1 *vs* 2 and 3
	198.7	44.4	174.8	36.6	166.2	32.9	<0.001	2 *vs* 1
								3 *vs* 1
**HDL (mg/dl)**								-
	59.4	14.9	57.3	13.0	52.9	12.8	0.033	-
								-
**Triglycerides (mg/dl)**								1 *vs* 3
	87.9	35.9	96.5	44.7	110.0	47.3	0.032	-
								3 *vs* 1
								1 *vs* 2 and 3
**LDL**	123.4	35.7	95.9	29.7	92.1	29.3	<0.001	2 *vs* 1
								3 *vs* 1
**Drugs taken (n.)**								1 *vs* 3
	5.3	2.8	6.4	2.8	7.1	3.1	0.014	-
								3 *vs* 1

DM, Diabetes Mellitus; SD, Standard Deviation; BIA, Bioelectrical Impedance Analysis; BMI, Body Mass Index; WC, Waist Circumference; CC, Calf Circumference; AC, Arm Circumference; MNA, Mini Nutritional Assessment; HbA1c, glycated hemoglobin; MM, total Muscle Mass; ASM, Appendicular Skeletal Muscle Mass; TBW, Total Body Water; ECW, Extra-Cellular Water; HDL, High-Density Lipoproteins; LDL, Low-Density Lipoproteins

The non-DM group had lower values for WC (92.6 *vs*. 97.6 and 99.4 cm, p=0.005), bone mass (2.2 *vs*. 2.4 and 2.5 kg, p<0.001), MM (40.6 *vs*. 44.8 *vs* 46.7 kg, p<0.001), and ASM (17.1 *vs* 19.1 and 19.7 kg, p<0.001), that the DM group. On the other hand, grip strength (19.1 *vs*. 26.1 and 25.6 kg, p<0.001), SARC-F (3.7 *vs*. 1.5 and 1.9, p<0.001), total cholesterol (198.7 *vs*. 174.8 and 166.2 mg/dl, p<0.001), and LDL (123.4 *vs* 95.9 *vs* 92.1 mg/dl, p<0.001), were higher in the non-DM group. Visceral fat (9.2 *vs*. 11.2 kg, p=0.010) and triglycerides (87.9 *vs*. 110.0 mg/dl, p=0.032) were significantly lower in the non-DM than uncontrolled DM, while no difference was found with respect to controlled-DM patients. Moreover, the total number of drugs taken was significantly lower in the non-DM than in the uncontrolled DM groups (5.3 *vs*. 7.1, p=0.014). Finally, the same trend was found in MNA scores (23.5 *vs*. 25.1, p<0.001).

To determine whether these variables could be independently associated with the presence of DM or its glycemic control, we conducted a stepwise multiple regression analysis (Table 3). Group membership was considered the dependent variable (non-DM: 0; controlled DM: 1; uncontrolled DM: 2), and various BIA-derived data, laboratory values, and the prevalence of diagnoses such as hypertension, dyslipidemia, metabolic syndrome, and sarcopenia were considered independent variables. LDL (coefficient -0.006, standard error 0.002, p=0.0100) was negatively associated with the dependent variable, while bone mass (coefficient 0.498, standard error 0.169, p=0.0042) was positively associated with the dependent variable. The other variables were excluded by the model (p>0.01).

**Table 3:** Multiple Regression – stepwise (y=groups)

Variable *	Coefficient	Standard Error	t	r partial	p
Bone mass (kg)	0.498	0.169	2.934	0.283	0.0042
LDL	-0.006	0.002	-2.627	-0.255	0.0100

* p>0.01 excluded by the model; LDL: Low-Density Lipoproteins

## DISCUSSION

Multidimensional assessment is one of the specific tools designed to assess older people suffering from several diseases [15]. Among these, conditions like DM and sarcopenia are becoming increasingly common among aging populations [5, 36]. Bioelectrical impedance is used to measure body composition and can help characterize these conditions, although there is currently no BIA validation in the DM sarcopenic population [36]. Our study aimed to determine the distribution of BIA-derived data among a group of non-DM people and two groups of controlled and uncontrolled DM people and to establish the independent association between them, lipidic asset, and the prevalence of metabolic syndromes with DM.

Our sample was divided into three groups according to the presence of DM and, where present, to its glucometabolic control, using HbA1c 7.0% as a discriminating cut-off level [48]. The sample was subjected to BIA, and the first surprising data that emerged was the wide variability of the BIA-derived data among the groups [36, 49]. Prior research highlighted the utility of BIA in diabetes management due to its non-invasive nature, allowing healthcare professionals to understand better how the disease affects patients' bodies and make more informed decisions about their treatment plans. Furthermore, patients with DM tend to show poorer performances than controls [36, 49]. In our sample, some variables were significantly better in the DM than the non-DM group, such as muscular and bone mass, representing a typical example of the higher sarcopenic risk in DM. Despite the lack of a universal global definition of sarcopenia, much less of sarcopenic risk [38, 50, 51], various scientific societies emphasize the critical importance of considering reduced muscular mass to define it [52, 53]. Moreover, there is a specific interest in the literature on managing sarcopenia in older patients with DM [54]. Several BIA-derived parameters, including increased visceral fat, along with factors like age, disease duration, and DM-related complications, are considered risk factors for sarcopenia. [55]. We observed a similar pattern in our sample with regard to serum lipid profiles, where both total cholesterol and LDL levels were better in DM patients. Additionally, the assessment of nutritional status and sarcopenic risk indicated that individuals with DM appeared to have better nutritional status and a lower risk of sarcopenia. These findings differ from the literature, which often reports a high prevalence of dyslipidemia among individuals with DM [56], even with a commonly found normal plasma LDL [57]. However, there may be a reasonable explanation for this discrepancy. Clinical trials and scientific studies typically exclude older individuals with complex medical histories, as the burden of comorbidities and polypharmacotherapy makes it challenging. Our outpatient service, specifically devoted to such a particular population, offers data from real-world experiences since frailty can overturn the paradigms usually studied. Moreover, the fact that DM patients had better physical performances and bioelectrical patterns can be explained by the fact that they had been visited by diabetologists, cardiologists, and/or nutritionists before our evaluation due to DM. In contrast, people without DM are less accurately followed by physicians since their cardiometabolic risk is widely considered lower. This aspect is also reflected by the lower number of drugs taken by non-DM patients in our study, consistent with the literature [58]. To provide a comprehensive perspective, it is widely known that cardiology and geriatrics play significant roles not only in the management of DM but also in addressing its prodromal stages [59-61].

In order to deepen our results, we performed a multivariate analysis to reduce the impact of covariates. While LDL levels reached statistical significance, their clinical impact was limited due to low coefficients. Bone mass showed a positive association with glucometabolic control, indicating that its values/incidence tend to increase in the presence of uncontrolled diabetes mellitus.

## CONCLUSION

In conclusion, we demonstrated that BIA may not be the ideal tool to discriminate between DM and non-DM elderly subjects since it can be influenced by a large number of covariates. Finally, a higher bone mass and lower LDL levels were independently associated with controlled and uncontrolled DM. The major strength of the study was that it examined a wide range of measurements obtained through BIA, providing a multidimensional perspective, which is fundamental to assessing elderly people. However, it also presents some limitations. Firstly, the study is monocentric, which may limit its generalizability to the broader geriatric population. Secondly, it does not take into account specific pharmacological classes of drugs despite their potential influence on metabolic and general health status. Lastly, the study evaluated patients without considering potential changes in body composition that could have been studied through a longitudinal design.

## Data Availability

The data and materials used and/or analyzed during the current study are not publicly available. These are available from the corresponding author upon reasonable request.
